# A Biological Micro Actuator: Graded and Closed-Loop Control of Insect Leg Motion by Electrical Stimulation of Muscles

**DOI:** 10.1371/journal.pone.0105389

**Published:** 2014-08-20

**Authors:** Feng Cao, Chao Zhang, Tat Thang Vo Doan, Yao Li, Daniyal Haider Sangi, Jie Sheng Koh, Ngoc Anh Huynh, Mohamed Fareez Bin Aziz, Hao Yu Choo, Kazuo Ikeda, Pieter Abbeel, Michel M. Maharbiz, Hirotaka Sato

**Affiliations:** 1 School of Mechanical and Aerospace Engineering, Nanyang Technological University, Singapore; 2 School of Electrical and Electronics Engineering, Nanyang Technological University, Singapore; 3 Division of Neurosciences, City of Hope Medical Center, Duarte, California, United States of America; 4 Department of Electrical Engineering and Computer Science, University of California, Berkeley, California, United States of America; University of Tours, France

## Abstract

In this study, a biological microactuator was demonstrated by closed-loop motion control of the front leg of an insect (*Mecynorrhina torquata*, beetle) via electrical stimulation of the leg muscles. The three antagonistic pairs of muscle groups in the front leg enabled the actuator to have three degrees of freedom: protraction/retraction, levation/depression, and extension/flexion. We observed that the threshold amplitude (voltage) required to elicit leg motions was approximately 1.0 V; thus, we fixed the stimulation amplitude at 1.5 V to ensure a muscle response. The leg motions were finely graded by alternation of the stimulation frequencies: higher stimulation frequencies elicited larger leg angular displacement. A closed-loop control system was then developed, where the stimulation frequency was the manipulated variable for leg-muscle stimulation (output from the final control element to the leg muscle) and the angular displacement of the leg motion was the system response. This closed-loop control system, with an optimized proportional gain and update time, regulated the leg to set at predetermined angular positions. The average electrical stimulation power consumption per muscle group was 148 µW. These findings related to and demonstrations of the leg motion control offer promise for the future development of a reliable, low-power, biological legged machine (i.e., an insect–machine hybrid legged robot).

## Introduction

Miniature legged robots are transportable, inconspicuous, and can pass through tiny openings and narrow corridors, which makes them excellent navigators for search and rescue missions at disaster sites. However, even state-of-the-art miniature legged robots have not yet matched the walking system of insects (ideal miniature legged systems in nature) in terms of power efficiency and motion controllability, despite many of them being inspired by the locomotion systems of living insects [1]–[3].

What if a living insect itself could be controlled using an electrical stimulator? That is, what if we could create an insect–machine hybrid robot—a fusion of a living insect and a man-made device (electrical stimulator)—to elicit our desired motions and behaviors? Compared with the power consumption in entirely man-made miniature legged robots [3], which is on the order of 100–1000 mW, that of an insect–machine hybrid robot can be drastically reduced because electrical stimulation consumes power on the order of only a few hundred microwatts [4]. Furthermore, insect–machine hybrid robots can be self-powered by energy harvesters, such as implantable biofuel cells, implanted into the living insect platform [5]–[10]. In addition, these robots can be more robust in motion control than robots that are entirely man-made. Complicated algorithms and controls, which are necessary for entirely man-made robots to retain their postures or to avoid or overcome obstacles, would not be necessary for insect–machine hybrid robots because the insect's intrinsic control system can be utilized if needed. For example, when an insect–machine hybrid robot encounters an obstacle, the user shuts off the electrical stimulator and releases the insect from the user's control system to allow it to avoid or overcome the obstacle by itself. Hence, overall, insect–machine hybrid robots would exhibit high power efficiency and excellent motion controllability.

Research related to insect–machine hybrid robots should be advanced from open-loop control systems to closed-loop control systems to allow these robots' legs to be regulated to follow the operator's predetermined motion path. Numerous research groups have investigated locomotion control or appendage motion response of various insects through electrical stimulation of insects' brain, ganglia, and nerve cords or muscles [11]–[25]. They have developed protocols and methodologies of electrical stimulation of neurons and/or muscles to elicit desired motion and overall locomotion (e.g., left–right turns in walking or in flight) in an open-loop control (i.e., non-feedback control) manner [12]–[18]. To date, researchers have focused on the development of stimulation protocols, i.e., determining what stimulation applied to which neuromuscular sites can elicit the desired motor actions and behaviors and evaluating the success rate and power consumption. Consequently, various stimulation protocols have been developed, but they have been demonstrated in an open-loop control manner. The questions arise as to what the next step should be in the research into insect–machine hybrid robots and how they can be practically used. Note that because of animals' intrinsically complicated motion control systems and the unavoidable physiological differences between individual animals, the elicited motions and behaviors vary from trial to trial and from animal to animal, even under identical stimulation protocols. As such, open-loop control techniques are insufficient for achieving precisely controlled motions; thus, closed-loop control must be introduced to reduce the deviations between the actually elicited motion and the user's predetermined and desired motion. Therefore, the next stage is the introduction of closed-loop control techniques to regulate insect legs to follow predetermined angular positions.

When a closed-loop system is used with an insect–machine hybrid robot to control, for example, an insect leg actuator, the electrical stimulation must have two critical functions: (1) reliably control the direction of elicited leg displacement and (2) induce a graded response in the magnitude of the leg displacement to different electrical stimuli (e.g., different stimulation frequencies and amplitudes). Reliability in the direction of elicited leg displacement, as defined in this study, means that we can elicit leg motion in the desired direction at a success rate of almost 100%, even though the elicited magnitude of the leg displacement exhibits some variation. To achieve such reliability in the displacement direction, we stimulated leg muscles instead of neurons. Neurons are densely arrayed and stacked, which makes the separate stimulation of individual neurons difficult. Even if an implanted electrode is strongly fixed at the targeted neurons, small drifts of the electrode on the order of the size of a single neuron would be impossible to avoid, and even a tiny drift of the electrode could cause undesired motor action at unexpected muscles. Compared with neurons, muscles are easier to visually identify and are sufficiently large to be observed under a conventional optical microscope. Variation in the position of an implanted electrode within the target muscle and/or drift of the implanted electrode can cause small changes in the elicited motion magnitude but might not affect the displacement direction. As such, we expected to achieve a high success rate in eliciting leg motion in the desired direction by stimulating leg muscles (see Results and Discussion).

With respect to the other key operation needed for a closed-loop control, (2) graded response, some variables must be identified to grade the magnitude of leg displacement elicited by electrical stimulation. Such variables can be used as manipulated variables in a closed-loop control system. Suppose a leg is displaced by electrical stimulation of a leg muscle in the desired direction, but the magnitude of the displacement is smaller than our predetermined value. If we know a specific variable that governs the magnitude to be graded, such as a higher value of the variable inducing a greater magnitude of leg displacement, we update the electrical stimulation to generate a higher value of that variable and output (apply) the updated stimulation to the muscle. For many insect muscles, muscle contraction is enhanced by increasing the rate of neural input (monitored as a spike in an electromyogram (EMG)) or increasing the electrical stimulation frequency (summation or facilitation) [18]–[22]. Thus, the electrical stimulation frequency can be used as a manipulated variable to grade the magnitude of leg displacement and as an output from the final control element to the leg muscle in a closed-loop control system. We measured the displacement of leg motion elicited by various stimulation frequencies. We also measured the EMG of a leg muscle group and associated it with the leg displacement. We then confirmed the tendency for both a higher neural input rate and a higher stimulation frequency to elicit a larger-magnitude leg displacement, and consequently decided to use the stimulation frequency as the manipulated variable in our closed-loop control system.

**Figure 1:**
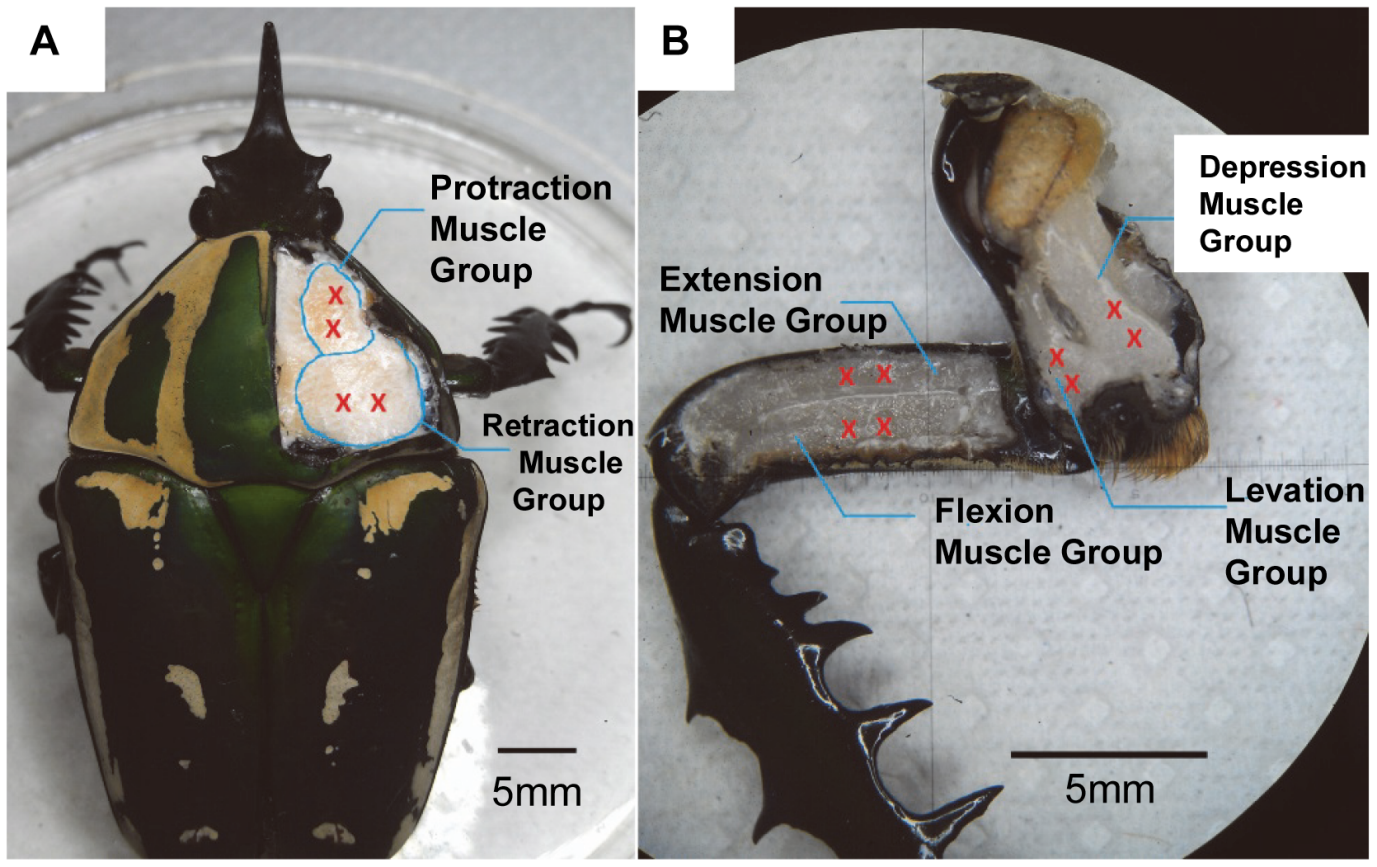
Anatomical view of a beetle's front leg. Anatomical view of the three pairs of antagonistic muscle groups that control a beetle's front leg. Red crosses indicate the implantation sites for stimulation electrodes. (A) The protraction/retraction muscle groups are inside the prothorax, connect the coxa to the pronotum, and control the protraction/retraction motion of the coxa. (B) The levation/depression muscle groups are inside the coxa and control the levation/depression motion of the femur. The extension/flexion muscle groups are inside the femur and control the extension/flexion motion of the tibia.

Overall, this paper reports the control of an insect's front leg motion by electrically stimulating multiple leg muscle groups in a closed-loop control manner. We successfully demonstrated a reliable biological microactuator with multiple degrees of freedom (DoFs). The three pairs of antagonistic muscle groups (Figure 1) of the insect leg enable the leg to have three DoFs: protraction/retraction, levation/depression, and extension/flexion. The threshold stimulation voltage to elicit significant leg displacement in the desired direction was determined. We then observed that the electrical stimulation frequency is a variable that governs graded leg motion (i.e., the magnitude of leg angular displacement) by measuring the elicited leg angular displacement and velocity at various stimulation frequencies and the muscle EMG associated with the leg motion. We then developed a closed-loop control system in which the stimulation frequency was the manipulated variable from the final control element to the leg muscle and the angular displacement of the leg was the system response. This closed-loop control system can regulate the leg to set at predetermined angular positions. Because the muscle configurations are similar among all the six legs of an insect, the successful motion control of the front leg will aid in developing motion controls for all other legs and overall walking control in an insect–machine hybrid robot in the future.

## Materials and Methods

### Study animal

We used the beetle *Mecynorrhina torquata* (order Coleoptera; length: 62±8 mm; mass: 7.7±1.9 g for all the beetles used in the experiments. Unless otherwise stated, all data are represented as mean ± standard deviation) as our insect platform for a biological actuator. The beetles were kept in separate plastic terrariums (20 cm×15 cm×15 cm) with woodchips at the bottom. They were fed sugar jelly every 2–3 days. The temperature and relative humidity in the terrariums were maintained at 25°C and 60%, respectively [4]. The use of this animal is permitted by the Agri-Food and Veterinary Authority of Singapore (AVA, HS code: 01069000, Product code: ALV002). Invertebrates, including insects, are exempt from ethics approval for animal experimentation according to the National Advisory Committee for Laboratory Animal Research (NACLAR) guidelines.

### Electrode implantation

A beetle was anesthetized by placing it in a small plastic zip bag filled with CO_2_ gas for 1 min. For immobilization, the beetle was subsequently placed onto a plastic or wooden plate and wrapped with dental wax (Cavex, Set Up Modeling Wax), which had been softened in hot water (80°C) for 10 s. Four small holes were made on its pronotum (positions indicated by red crosses in Figure 1A) using an insect pin (Indigo Instruments, enamel-coated #5). Using the same technique, eight more holes were made on the coxa and femur (positions indicated by red crosses in Figure 1B). A thin Teflon-insulated silver wire (A-M Systems, 127 µm uncoated diameter, 178 µm Teflon-coated diameter) was used as the stimulating electrode. The insertion depth of the electrode was 2 mm from the outer surface of the cuticle. Both ends of the silver wire were heated in a flame to remove the insulation and enable electrical contact at the ends.

### Electrical stimulation

To obtain a suitable threshold voltage, the non-implanted end of the wire was connected to the output channel of a function generator (Agilent, 33220A). The stimulation pulse width was fixed at 1 ms and the frequency was fixed at 30 Hz. The stimulation voltage was varied in increments of 0.25 V, starting at an initial stimulation voltage of 0.25 V.

To investigate the elicited leg motion due to different stimulation frequencies, the three motion types of protraction/retraction, levation/depression, and extension/flexion were analyzed individually. For example, when investigating the protraction/retraction response to different stimulation frequencies, we restricted the levation/depression and extension/flexion motions by inserting an insect pin into the corresponding articulation. Two markers placed on the beetle's leg were recognized by a 3D motion capture system as a solid line segment, and the third marker placed on the beetle's body indicated the beetle's position. The 3D motion capture system recognized and stored the *X*, *Y*, and *Z* coordinates of all markers. Angular displacement was determined using the following formula for calculating the angle between two vectors:
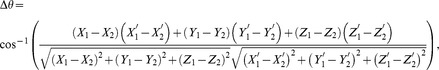
where *X*_1_, *Y*_1_, and *Z*_1_ and *X*_2_, *Y*_2_, and *Z*_2_ are the initial coordinates of markers 1 and 2, respectively, and *X*_1_′, *Y*_1_′, and *Z*_1_′ and *X*_2_′, *Y*_2_′, and *Z*_2_′ are the coordinates of the two markers as a consequence of the beetle's leg motion. Therefore, all angular displacement values were calculated with respect to the leg's initial (resting) position. The position of each leg segment at rest (before electrical stimulation) was defined as the initial position (the initial position varies from beetle to beetle; the variation is on the order of a few degrees). Each time after the leg muscle was stimulated, we manually positioned the leg to its initial position by checking the 3D coordinates of the markers placed on the beetle's leg. For all experiments, the stimulation voltage was fixed at 1.5 V and the pulse width was fixed at 1 ms.

The electrical stimulation power consumption was measured for all six muscle groups present in the beetle's front leg. Current flow through a muscle was measured using an oscilloscope (Yokogawa, DL 1640), and a function generator (Agilent, 33220A) was used to supply a positive pulse train at 100 Hz, 1.5 V, and a 1-ms pulse width.

### Measurement of leg-muscle EMGs synchronized with leg motion

A pair of thin silver wires (A-M Systems, 127 µm uncoated diameter, 178 µm Teflon-coated diameter) were implanted into the muscle group of interest using the technique described in the electrode implantation section. The electrodes were glued to the outer surface of the cuticle with dental wax (Cavex, Set Up Modeling Wax) to avoid potential artifacts due to the electrodes' drift. The EMG signals were amplified 500-fold using an amplifier (LT1920, Burr-Brown Products). A custom-programmed wireless microprocessor (Texas Instruments, CC2431, 6×6 mm^2^, 130 mg, and 32 MHz clock) was used to collect the EMG signals from the muscles at a sampling rate of 2000 Hz. The input/output (I/O) pins of the microprocessor were set at inputs so that EMG signals from the muscles were collected as input electrical potentials. The beetle's leg motion was captured in the same manner as that described in the previous section (“Electrical stimulation”) using the 3D motion capturing system. We developed a customized software tool, BeetleCommanderEMG, which can simultaneously collect and store EMG signals from the microprocessor and the beetle's leg motion information from the 3D motion capturing system. The threshold voltage of the EMG signal was determined individually for each experimental result to identify the maximum number of EMG spikes captured [26], [27]. The EMG burst onset time was defined as the time at which the voltage of an EMG spike exceeds a certain threshold value. The EMG burst termination time was defined as the end time of the last detectable EMG spike. The mean EMG frequency was calculated as the average of the instantaneous frequencies within a single burst. The average angular velocity was calculated as the linear regression slope of the angular displacement during the time interval of motion. The onset time of leg motion was defined as the time of first detectable leg retraction motion. The motion offset time was defined as the beginning motion of the first detectable protraction motion [26], [27].

### Closed-loop control system

**Figure 2:**
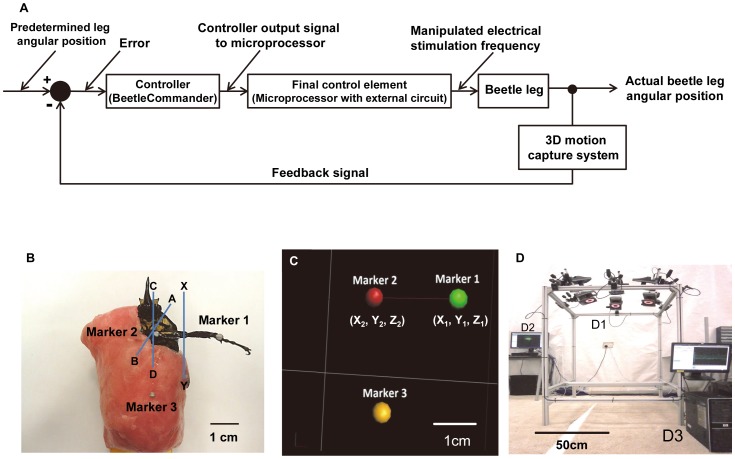
Schematic representation of the closed-loop control system and markers captured by a motion capture system. (A) Schematic of the closed-loop control system. Instantaneous marker positions are displayed and used as the feedback information for stimulation frequency adjustments. (B) Three 2-mm-diameter reflective markers placed on a beetle for motion capture purposes. *A*–*B* is the axis of rotation of the protraction/retraction motion of the coxa, *C*–*D* is the axis of rotation of the levation/depression motion of the femur, and *X*–*Y* is the axis of rotation of the extension/flexion motion of the tibia. (C) Markers placed on the beetle are recognized by the 3D motion capture system as point objects and displayed on a computer screen. Two markers placed on the beetle's front leg were recognized as a solid line segment that represented the femur–tibia section of a beetle's leg, and the third marker on a beetle's body indicated the beetle's body position. (D) Overview of the 3D motion capture system. This 3D motion capture system captures and stores the *X*, *Y*, and *Z* coordinates of all markers. The stored 3D marker positions were used for precise numerical analyses of a beetle's leg motion. The closed-loop control system calculated the instantaneous angular displacements of leg motion and used this information to adjust the output stimulation frequencies. (D1) The system comprised six T40s VICON cameras (with resolution of 4 megapixels (2336×1728)) operating at 100 frames per second. (D2) A VICON server was used to acquire the camera signals and construct the marker positions in real time. (D3) A computer was used to collect the marker-position data from the server and issue stimulation commands to the final control element.

We developed a closed-loop motion control system (Figure 2A) to be introduced into BeetleCommander. An electrical stimulation signal was generated using a custom-programmed microprocessor (Texas Instruments, CC2431, 6×6 mm^2^, 130 mg, 32 MHz clock). Electrical stimulation signals generated from two separate stimulation channels were used to control one pair of antagonistic muscle groups. The BeetleCommander system could extract instantaneous marker position information (Figures 2B and C) from the 3D motion capture system (Figure 2D) and calculate the immediate leg angular position. Update time intervals (i.e., the time interval at which the closed-loop system updated the instantaneous leg position and output stimulation frequency) were user-adjustable. The concept of proportional control was also used to adjust the magnitude of a step increment or decrement of the stimulation frequency:
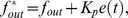
where 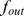 is the last output stimulation frequency from the final control element, 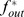 is the updated output stimulation frequency from the final control element (its value is limited to the 10–200 Hz range), 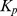 is the proportional gain (user-adjustable), and 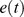 is the instantaneous angular displacement error at time *t*.

Protraction/retraction closed-loop motion control was used as an example of operation, which was controlled by two separate channels from the final control element. Electrical stimulation signals from the two channels were generated on the basis of predetermined angular positions set by the user. Both stimulation channels operated independently and concurrently. The electrical stimulation frequency from one channel was initially increased to elicit the leg to move to the desired position. If the actual leg angular position was greater than the predetermined angle, the stimulation from the current working channel started to decrease its stimulation frequency while that from the counter channel began to decrease the angular position by increasing the stimulation frequency. Likewise, if the actual angular position was less than the predetermined angle, the counter channel stopped generating its stimulation signal and the other working channel began to increase its stimulation frequency to increase the leg angular position. Therefore, the two channels used to stimulate the pair of antagonistic muscle groups operated simultaneously to ensure that the beetle's leg followed the predetermined angular positions.

## Results and Discussion

### Threshold stimulation voltage to elicit leg displacement

**Figure 3:**
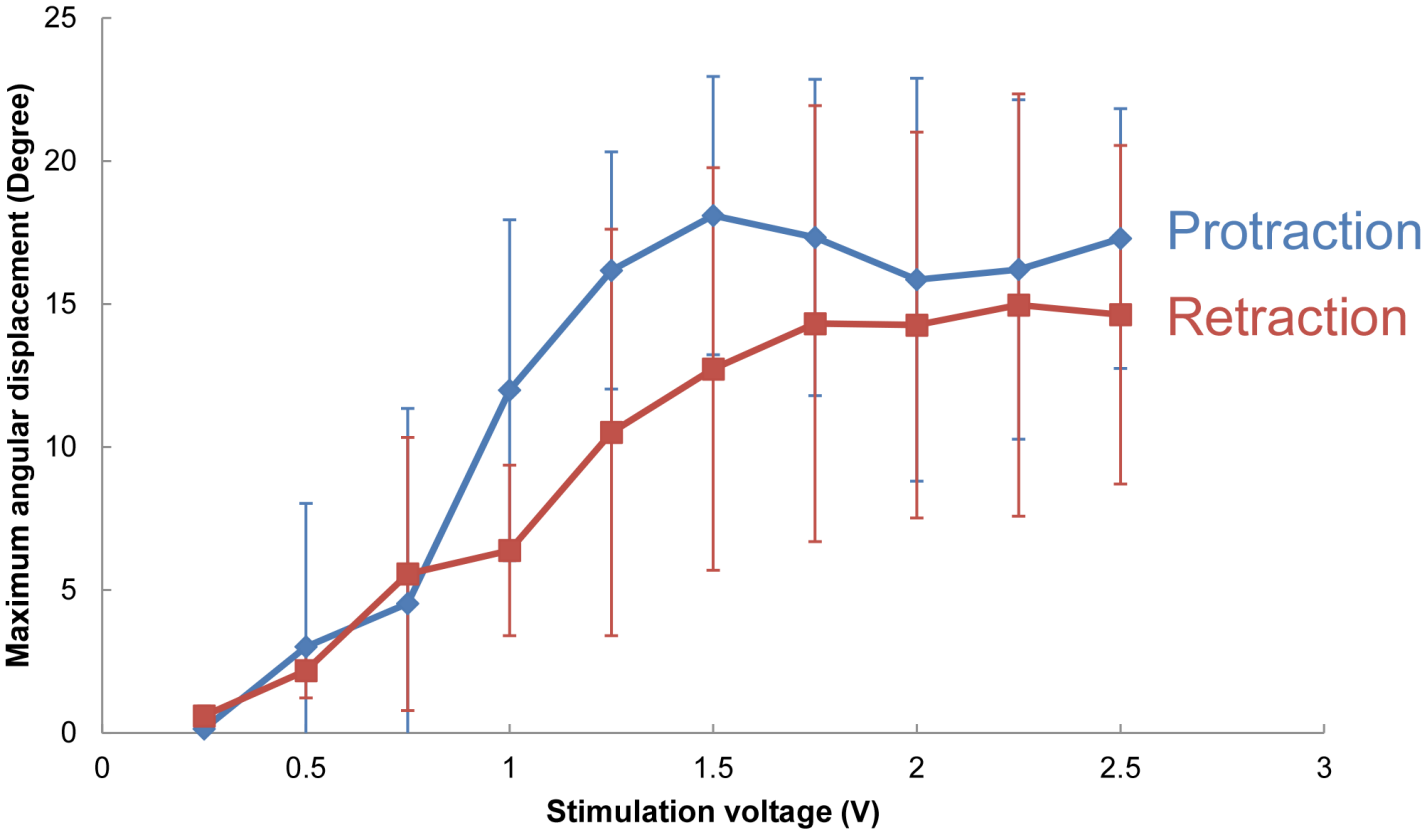
Maximum angular displacement elicited by stimulation amplitudes varied from 0.25 V to 2.5 V. A 30 Hz and 1 ms pulse width monophasic pulse train with varying amplitudes was used to determine the threshold voltage. Leg angular displacement (absolute values used for all the motions) occurred at approximately 1 V and increased steadily until 1.5 V (number of beetles = 5, 17≤ number of data points at each stimulation voltage ≤22). The angular displacement remained maximal when the stimulation voltage ranged from 1.5 to 2.5 V.

We fixed the stimulation frequency at 30 Hz and used a 1 ms monophasic pulse train with amplitudes ranging from 0.25 V to 2.50 V. Figure 3 shows the elicited protraction/retraction angular displacements at various stimulation voltages (number of beetles = 5 and 17≤ number of data points at each stimulation voltage ≤22). The threshold voltage required to elicit a beetle's leg movement was approximately 1.0 V, and the maximum angular displacement was reached at approximately 1.5 V (maximum protraction/retraction angle at 1.5 V = 18.09°±4.87°/12.73°±7.04°). When the stimulation voltage exceeded 1.5 V, the maximum angular displacement for both protraction and retraction remained relatively constant. For optimal results, we had to set the stimulation voltage as low as possible to minimize any possible damage to the beetle's muscle while simultaneously ensuring that the stimulation voltage was sufficiently high to reliably elicit the desired leg motion. Thus, we fixed the stimulation voltage at 1.5 V for all subsequent experiments. With a 1.5 V stimulation voltage, the success rate for inducing leg movement in the desired direction was 100% (number of beetles = 42). In addition, after repeatedly applying the electrical stimulation to a single muscle group more than 200 times within one day during a single experiment, we observed no obvious indications that the beetle's muscle was damaged by the 1.5 V stimulation voltage.

### Stimulation frequency as a variable to grade the leg displacement magnitude

**Figure 4:**
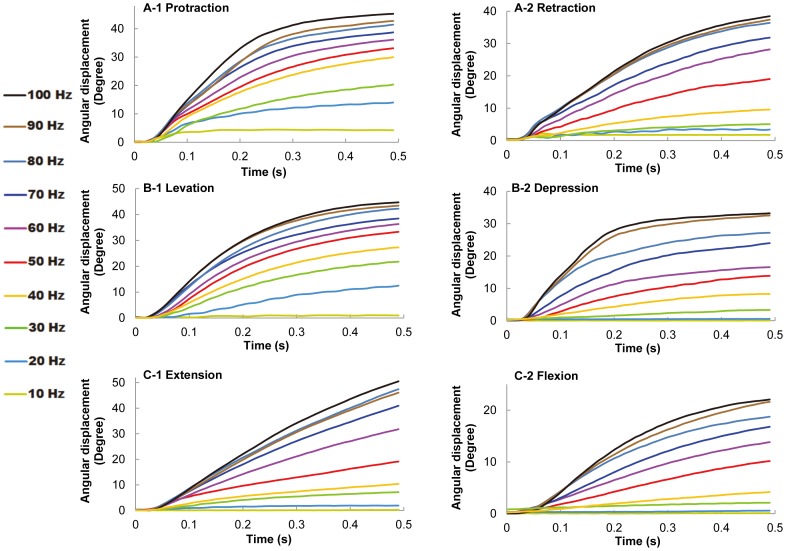
Demonstration of graded leg motion control. (A–C) Angular displacement profiles (absolute values used for all the motions) of (A) protraction/retraction, (B) levation/depression, and (C) extension/flexion motions elicited at stimulation frequencies ranging from 10 to 100 Hz. The maximum angular displacement increased as the stimulation frequency was increased for all motion types. Furthermore, the slope of the angular displacement curve also increased with increasing stimulation frequency, which suggested that higher stimulation frequencies elicited greater angular velocities and that greater force was therefore elicited in the beetle's leg.

**Figure 5:**
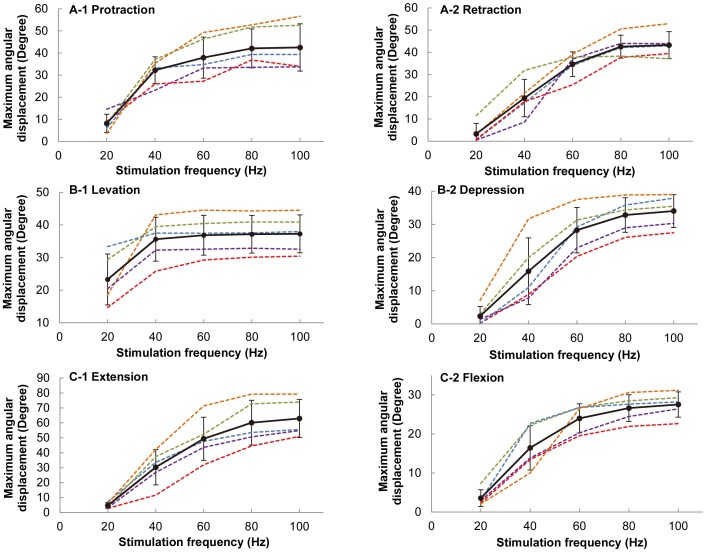
Maximum angular displacement as a function of stimulation frequency for all six motion types. (A) protraction/retraction, (B) levation/depression, and (C) extension/flexion elicited at stimulation frequencies ranging from 20 to 100 Hz at step increments of 20 Hz. The different colors of dotted lines indicate different beetles used in the experiments. The thick black line represents the average maximum angular displacement (absolute values used for all the motions) at various stimulation frequencies (number of beetles = 5, number of data points at each stimulation frequency  = 25 for each motion type).

**Figure 6:**
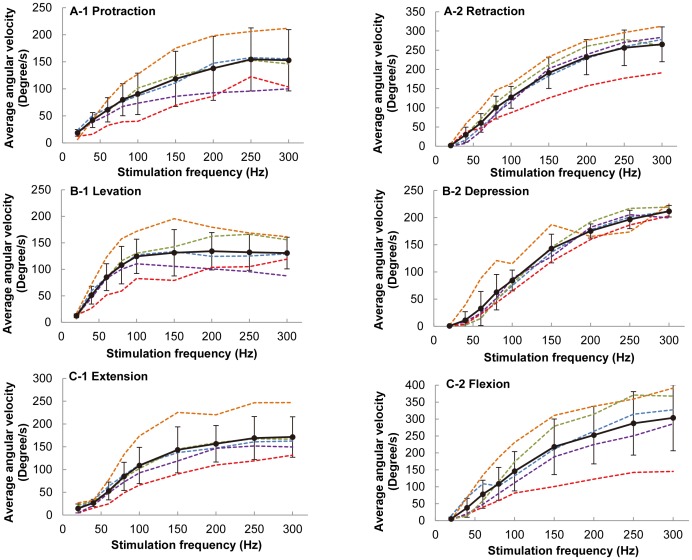
Average angular velocity as a function of stimulation frequency for six motion types. (A) protraction/retraction, (B) levation/depression, and (C) extension/flexion elicited at stimulation frequencies ranging from 20 to 300 Hz at a step increment of 20 Hz at frequencies less than 100 Hz and at a step increment of 50 Hz at frequencies greater than 100 Hz. The different colors of dotted lines indicate different beetles used in the experiments. The thick black line represents the mean value of the average angular velocity (absolute values used for all the motions) at various stimulation frequencies (number of beetles  = 5, number of data points at each stimulation frequency  = 25 for each motion type).

For all experiments, the stimulation voltage was fixed at 1.5 V and the pulse width was fixed at 1 ms. The elicited leg motion at various stimulation frequencies was studied. As shown in Figure 4, the resulting angular displacements for all six motion types monotonically increased with the stimulation frequency (number of beetles = 10). The angular displacement of the beetle's leg reached its limit when the stimulation frequency exceeded approximately 80 Hz for the other five motion types, with the exception of levation motion (Figure 5; the maximum angular displacement for levation was reached at stimulation frequencies of approximately 40 Hz). This maximum angular displacement might be due to the mechanical limitation of the beetle's leg structure. Similarly, the average angular velocity also increased monotonically with the stimulation frequency (Figure 6). When the stimulation frequency was greater than 250 Hz, the average angular velocity of the leg motion reached the maximum value for the other five motion types, with the exception of levation motion (Figure 6, maximum average angular velocity of levation was reached at a stimulation frequency of approximately 100 Hz).

### Comparison between muscle stimulation and sensory system stimulation

As discussed in the Introduction, the performance of a closed-loop control system in stimulating a muscle is compared to that of a sensory system (e.g., a system stimulated by an antenna or compound eye) on the basis of two key performance requirements: (1) reliability in the direction of elicited leg displacement and (2) a graded response in the leg displacement magnitude. As demonstrated in the previous two sections, our leg-muscle stimulation satisfies both these key requirements. However, numerous researchers have demonstrated various protocols of stimulating sensory systems to control insect locomotion in walking and in flight [15]–[18]. For instance, Holzer and Shimoyama [16] stimulated the antennae of a cockroach (*Periplaneta americana*) to control its walking direction. Transient (100–200 ms) turning behavior was observed when the ipsilateral antenna of a walking cockroach was stimulated. However, the electrodes used to stimulate sensory systems are not well secured, and the two aforementioned performance requirements are not guaranteed. Unlike muscle stimulation, the stimulation of sensory systems does not result in a 100% success rate in eliciting the desired motor action or behavior. A graded response in a target muscle by stimulation of sensory systems is possible and has actually been demonstrated [16], however, this approach is relatively difficult and less reliable than muscle stimulation, as discussed in the Introduction. Overall, the adaptation of muscle stimulation in closed-loop motion control is preferred over the adaptation of sensory system stimulation.

### Comparison between the leg motions elicited by electrical stimulation and those by intrinsic neural input

**Figure 7:**
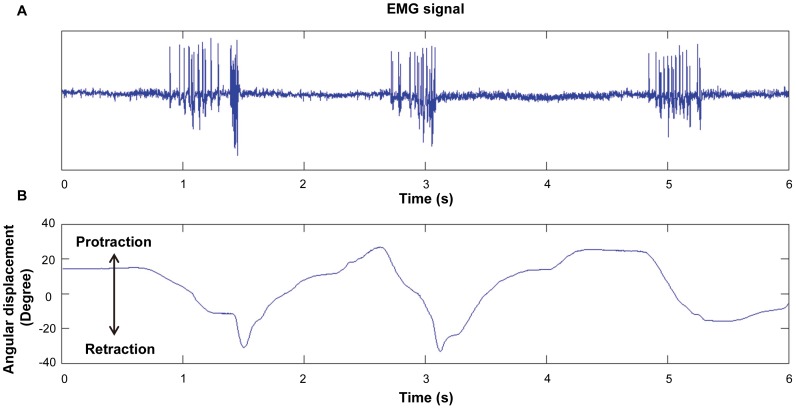
A typical retraction muscle EMG signal vs. time synchronized with beetle's front leg protraction/retraction motion. (A) The EMG signal from the retraction muscle group synchronized with (B) the front leg's protraction/retraction motion. Decrease in the angular displacement represents retraction motion of the front leg. No obvious EMG signals were observed when the beetle was at rest or when it performed protraction motions.

**Figure 8:**
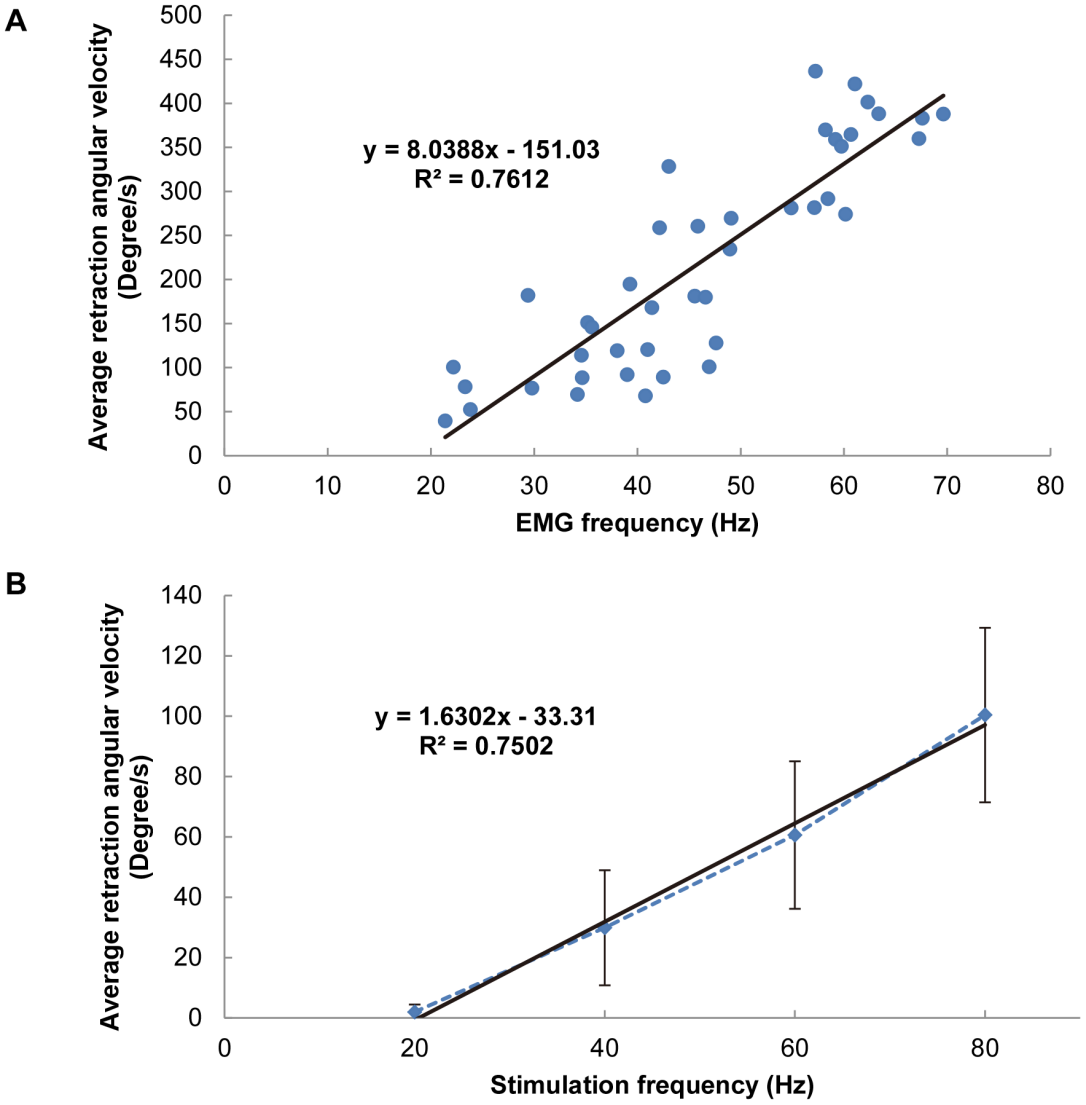
Average retraction angular velocity as functions of average muscle EMG frequency and electrical stimulation frequency. (A) Average retraction angular velocity vs. EMG frequency (number of beetles  = 4, total number of data points  = 43). (B) Average retraction angular velocity vs. electrical stimulation frequency (number of beetles  = 5, number of data points at each stimulation frequency  = 25). The black straight line in each graph is the least-squares linear regression line for the corresponding data. A significant linear relationship existed for both average retraction angular velocity vs. the average muscle EMG frequency (*R* ^2^ = 0.76) and the average retraction angular velocity vs. the electrical stimulation frequency (*R* ^2^ = 0.75).

To understand how effectively our electrical stimulation (synthetic input to muscle) mimics an animal's intrinsic neuromuscular system (natural input to muscle), we recorded the EMG signal of the retraction muscle group during natural leg motion and synchronized the EMG with the leg displacement (Figure 7). A decrease in the angular displacement corresponds to a retraction motion in the figure. Figure 8A shows the average retraction velocity as a function of the mean muscle EMG frequency (number of beetles = 4, total number of data points = 43). The maximum EMG frequency recorded was approximately 70 Hz. A linear relationship was observed between the angular velocity and the mean EMG frequency. Figure 8B shows a plot of the average retraction velocity of the beetle's front leg as a function of the electrical stimulation frequency (number of beetles = 5, number of data points at each stimulation frequency = 25). Another linear relationship is observed in this figure, which indicates that a higher stimulation frequency resulted in higher angular velocity and hence elicited greater muscular force [20]–[25]. Despite the expected result that the angular velocity monotonically increases with both average muscle EMG frequency and electrical stimulation frequency, the slope of the linear regression line for the former case (Figure 8A) is approximately five times steeper than that for the latter case (Figure 8B); that is, provided that the EMG frequency is the same as the electrical stimulation frequency, the resultant leg motion is five times faster when the beetle moves voluntarily compared with when it is electrically stimulated. This difference might be due to the basic differences between the EMG signal (natural neural input) and the electrical stimulation signal (synthetic input) in terms of amplitude and signal forms. In addition, because the beetle might have resisted the electrically elicited motion by activating the antagonist muscles and produced an opposing force, the angular velocity of leg motion elicited by electrical stimulation could have been smaller than that expected, on the basis of the tendency of the motion associated with the EMG spike frequency.

### Demonstration of a closed-loop control system for insect leg motion

**Figure 9:**
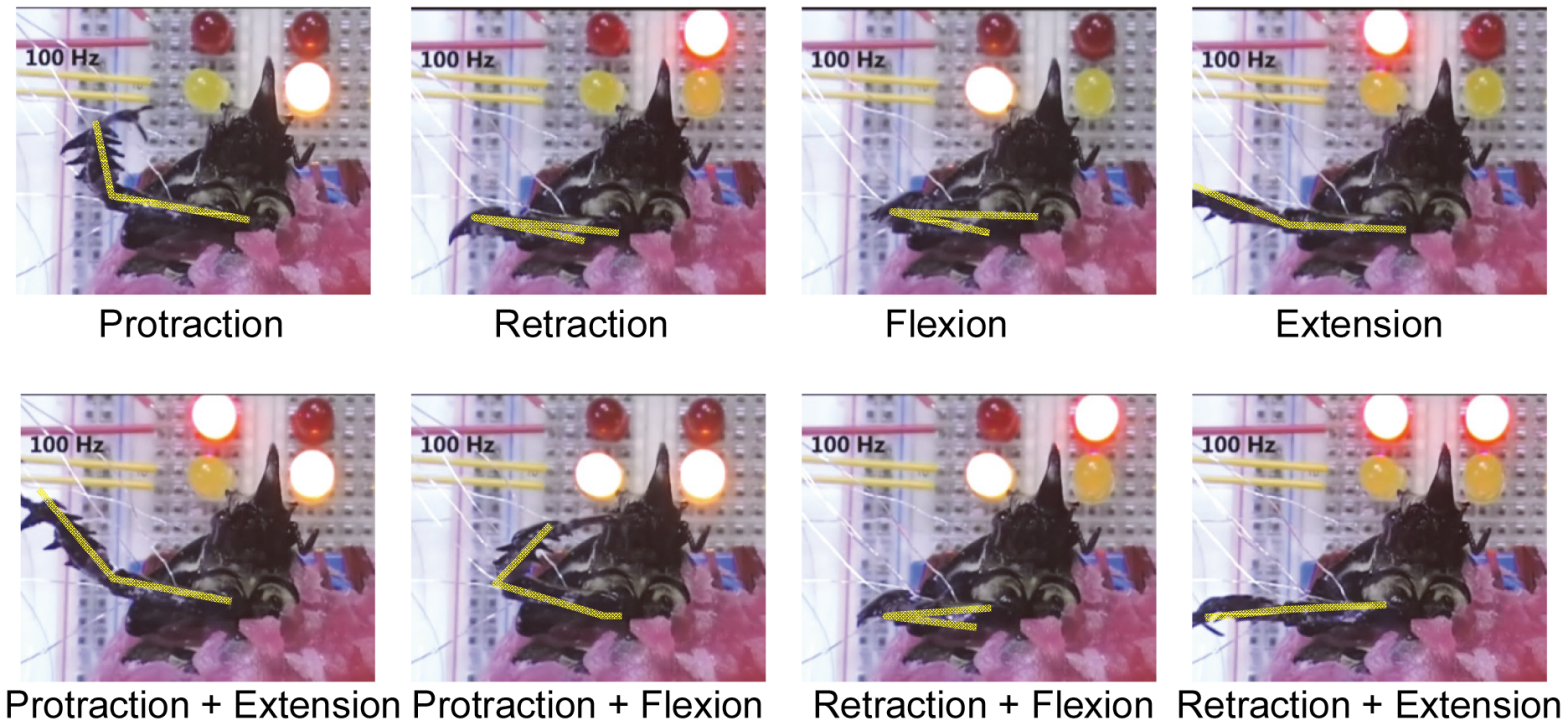
Demonstration of controlling a beetle's front leg motions by electrical stimulation of muscles. Protraction, retraction, flexion, and extension motions of a beetle's front leg elicited by electrical stimulation with a positive pulse train at 100 Hz and a 1 ms pulse width (as in Video S1). Each locomotion type was first stimulated individually, as shown in the upper four images; two muscles were then stimulated simultaneously to produce combined leg motion, as shown in the lower four images. Each light-emitting diode (LED) near the beetle's head indicated the time when a particular stimulation site had been switched on.

**Figure 10:**
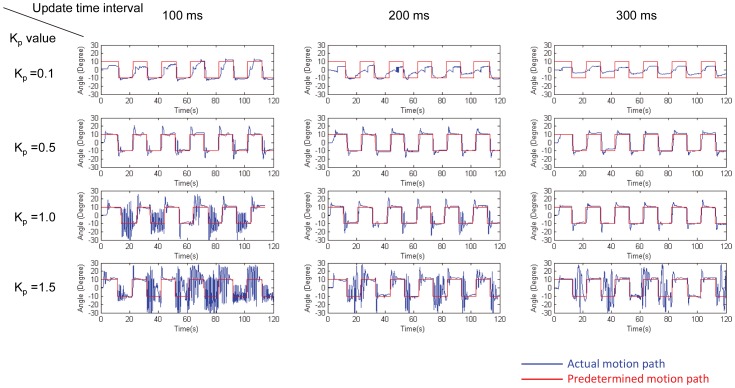
Representative closed-loop control of protraction/retraction motion at different *K*_p_ values and update time intervals. Comparison of the actual leg angular position (blue path) with a predetermined angular position (red path) during closed-loop control of protraction/retraction of a beetle's front leg at *K*_p_ = 0.1, 0.5, 1.0, and 1.5 and for update time intervals of 100 ms, 200 ms, and 300 ms. Positive angular displacement represents the retraction motion while negative angular displacement represents the protraction motion.

**Figure 11:**
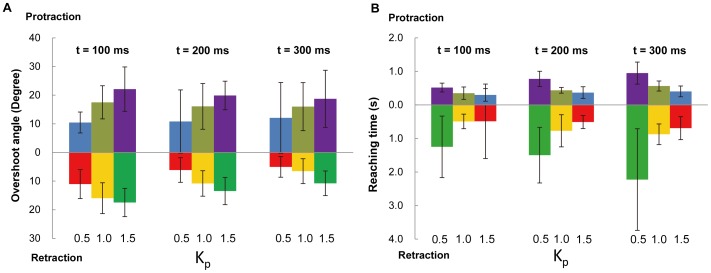
Variation in leg motion responses to different closed-loop control settings. (A) The overshoot angle and (B) the reaching time of protraction/retraction motion with respect to different *K*_p_ values and update time intervals *t* during closed-loop control (number of beetles = 5, 35≤ number of data points at each experiment setting ≤49). In general, as the *K*_p_ value was increased from 0.5 to 1.5 and the update time interval *t* was decreased from 300 ms to 100 ms, (A) the leg response overshoot angle (absolute values used for all the motions) increased, whereas (B) the reaching-time decreased. Numbers at the bottom of each graph indicate the *K*_p_ value used for the corresponding column above.

As each muscle group was stimulated by independent, isolated outputs from the stimulator board, we could elicit the individual leg motion types either separately or simultaneously, as demonstrated in Figure 9 and Video S1. Moreover, on the basis of the findings that the leg angular displacement monotonically increased with respect to the stimulation frequency, we developed a closed-loop control system to make the leg move according to preset angular positions. Figure 10 shows typical closed-loop control results (number of beetles = 5) for protraction/retraction motion; these results were obtained by comparing the actual leg movement achieved (blue path) with the predetermined leg angular position (red path) at *K*_p_ values of 0.1, 0.5, 1.0, and 1.5 and at update time intervals of 100, 200, and 300 ms (the update time interval is the time interval at which the closed-loop system updates the instantaneous leg position and output stimulation frequency). Figure 11 shows the overshoot and reaching time of the closed-loop control experiment (number of beetles = 5, 35≤ number of data points at each experiment setting ≤49). The statistical information for the experiments in which *K*_p_ = 0.1 is omitted from Figure 11 because the rate of increase of the muscle stimulation frequency is not always sufficient to bring the leg to the predetermined angular position due to the relatively small *K*_p_ value (see Figure 10, *K*_p_ = 0.1, update time interval = 100, 200, and 300 ms). Of the 243 experimental data points for *K*_p_ = 0.1, the beetle's leg reached the predetermined angular position 104 times (42.8% success rate). However, for *K*_p_ = 0.5, 1.0, and 1.5, the beetle's leg was always (100% success rate) brought to the predetermined angular position (Figure 10). As evident in Figure 11 and Tables S1 and S2, when the *K*_p_ values were increased, the leg response overshoot generally increased, whereas the reaching time (i.e., the time required to reach the predetermined angular position) decreased. For example, when the *K*_p_ value was increased from 0.5 to 1.5 using an update time of 100 ms, the protraction overshoot angle increased from 10.47°±3.66° to 22.12°±7.75° and the retraction overshoot angle increased from 11.03°±5.06° to 17.48°±4.94°; in contrast, the protraction reaching time decreased from 0.518±0.133 s to 0.299±0.188 s and the retraction reaching time decreased from 1.249±0.917 s to 0.488±1.111 s. This tendency was consistent because larger *K*_p_ values would result in greater changes in the output stimulation frequency (either increases or decreases) for the same instantaneous angular displacement error. This larger output stimulation frequency change would then elicit a greater angular displacement and a greater angular velocity of the beetle's leg (i.e., a larger force elicited in the muscle). As a result, the beetle's leg would move at a faster rate (decreased reaching time). However, at the same time, the beetle's leg would be more likely to exceed the predetermined angular position (increased overshoot). In general, as the update time interval was incrementally changed, the leg response overshoot decreased, whereas the reaching time increased (Figure 11, comparison across different *t* values, and Tables S1 and S2). For example, for *K*_p_ = 1.0, as the update time was increased from 100 to 300 ms, the protraction overshoot angle decreased from 17.51°±5.79° to 16.01°±8.39° and the retraction overshoot angle decreased from 15.97°±5.38° to 6.52°±4.34°, whereas the protraction reaching time increased from 0.350±0.184 s to 0.564±0.150 s and the retraction reaching time increased from 0.492±0.217 s to 0.875±0.308 s. This tendency was consistent because a larger update time interval would result in a longer required period for the beetle's leg to respond to the electrical stimulation. This longer response period decreased the likelihood that the 3D motion capture system would capture the leg position before it reached its final position. Therefore, this longer response period decreased the likelihood that the BeetleCommander closed-loop control system would overly increase or decrease the stimulation frequency, which would result in reduced overshoot. However, at the same time, with an increasing update time interval, the reaching time would increase.

The statistically obtained reaching times and overshoots at the different system settings (the proportional gain and update time interval) reveal certain constraints and limitations in the design of insect–machine hybrid legged robots in terms of the step cycle of the leg in the walking gait. We can refer to the reaching-time data to determine the appropriate stepping frequency (e.g., the duration of each step should be longer than the combined reaching time of all motions involved in that step). Given the angular overshoot, we can estimate the step-length error present in a given closed-loop control system. For example, when *K*_p_ and the update time interval *t* are set to 0.5 and 300 ms, respectively, the overshoot for retraction motion is 5.05° on average (Figure 11 and Table S1). This overshoot angle of 5.05° results in an estimated step-length error of approximately 0.22 cm if the beetle's leg length is assumed to be 2.50 cm. As such, even for future advanced close-loop control systems, we can refer to these indices (e.g., the reaching time and overshoot) to reduce the constraint and limitation of the step cycle and other relevant parameters in insect–machine hybrid legged robots.

### Potential experimental errors

The use of living organisms in experiments can introduce several unavoidable errors, which is why closed-loop motion control is necessary. For example, the neutral or resting position of the leg differs within a few degrees from beetle to beetle. Although some researchers have defined the neutral position of a joint in terms of the angular position between the two leg segments (e.g., Guschlbauer et al. [19] defined the neutral position of the femur-tibia joint of the stick insect as 90°), ensuring that the leg appendage of a living insect rests at the neutral position, as predefined by us, is difficult.

The CO_2_ anesthetization of the beetle before implantation of the stimulation wires (see the Electrode implantation section) can affect the leg response. Several side effects of CO_2_ anesthetization on insects have been reported [28]–[33]. CO_2_ anesthetization can increase haemolymph acidity and cause the heartbeat to stop [30], [33]. As a result, exposure to CO_2_ can impair oxygen delivery to tissues, thereby reducing oxidative phosphorylation and adenosine triphosphate (ATP) production in cell mitochondria [29]. These effects can significantly influence the efficacy of a bio-actuator and are known to affect insects' locomotion [28]. Nonetheless, CO_2_ exposure is one of the most popular anesthetic methods in entomological research [30]–[32], even though its side effects have not yet been fully elucidated [31]–[33]. Improved anesthetic methods might be helpful in future entomological research.

Despite these potential experimental errors, the magnitude of the leg displacement is undoubtedly increased by increasing the stimulation frequency (Figure 4). Inspired by this graded response to the stimulation frequency, we successfully developed a closed-loop control system to regulate the leg to set at predefined angular positions; this controlled response is independent of the potential experimental errors in leg response because closed-loop control systems, in general, reduce such errors.

### Power consumption

**Figure 12:**
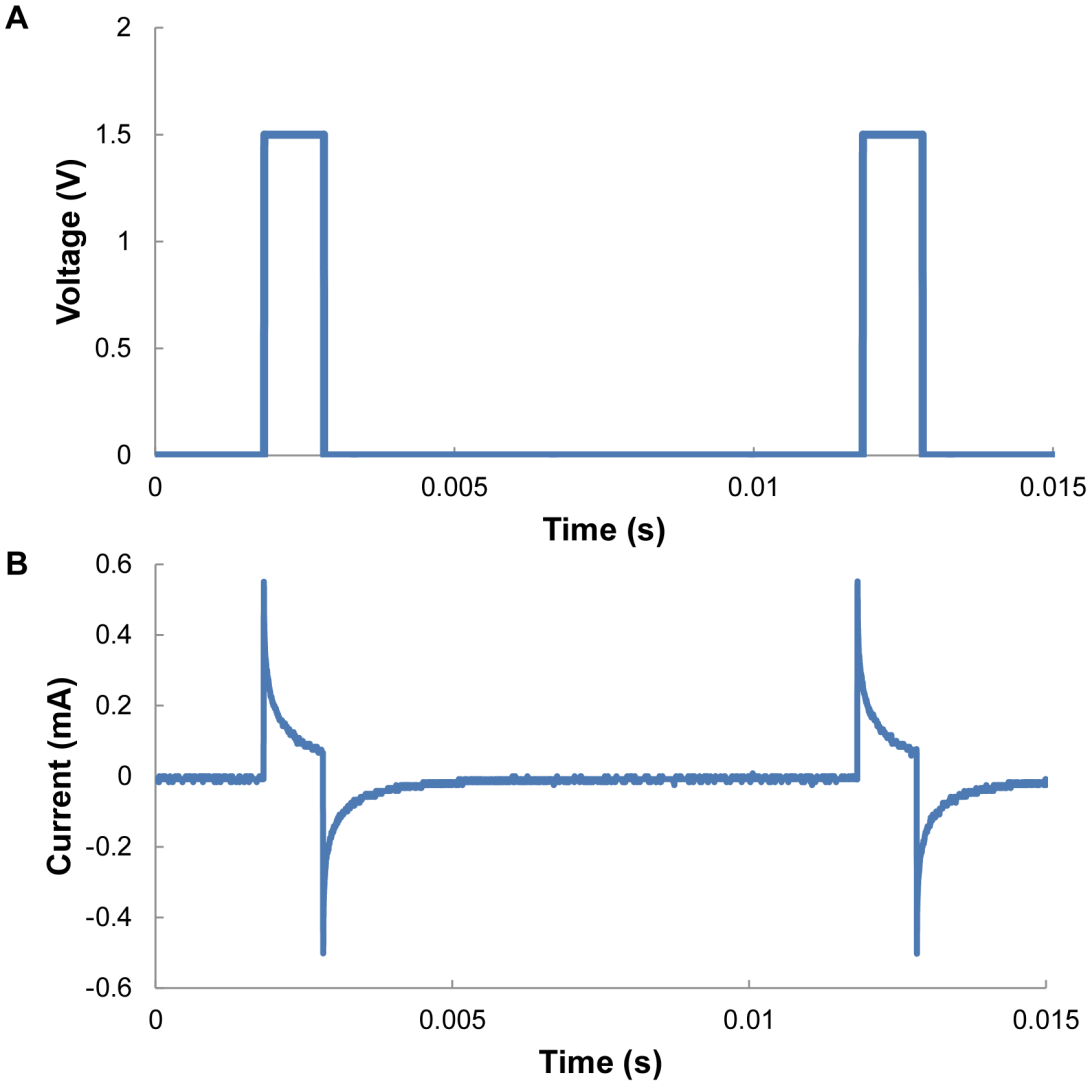
Representative stimulation pulses and electrical current flow through the retraction muscle group. (A) A positive stimulation pulse train of 1.5 V, 100 Hz, and a 1 ms pulse width produced by the function generator was applied between the two electrodes inserted in the retraction muscle group of a beetle's front leg. (B) Typical current wave that passed through the beetle's muscle (depression muscle group of the front leg).

We confirmed that the power consumption of the insect leg actuator was remarkably low (on the order of 100 µW to a few milliwatts). Figure 12A shows the positive pulse train at 100 Hz, 1.5 V, and a 1 ms pulse width used as the muscle stimulation signal. Figure 12B shows a typical current-flow profile through the depression muscle group. The power consumption of electrical stimulation on all six muscle groups of the front leg was measured across five different beetles. Table S3 shows the detailed numerical values of the mean and standard deviation in power consumption (number of beetles = 5, number of data points collected from each muscle group = 40). The average power consumption of the electrical stimulation of a single muscle group was 148 µW. Assuming that the stimulations of the middle and hind legs consume similar amounts of power, the power consumption of an insect–machine hybrid legged robot using a beetle is approximately 5.3 mW in the worst-case scenario, where all six muscle groups are stimulated simultaneously for all six legs (stimulation of 36 leg muscle groups). Note that the worst case scenario should be far from the actual case because just half of the muscle groups would be simulated simultaneously in actual insect walking control. Nonetheless, this power consumption is considerably low compared with the 100–1000 mW order of power consumption in entirely man-made miniature legged robots [3].

## Conclusions

The experimental results demonstrated that electrical stimulation with a threshold voltage of 1.5 V elicited significant displacement of the leg in desired directions (three DoFs, i.e., protraction/retraction, levation/depression, and extension/flexion) at a 100% success rate. The magnitude of the leg displacement was graded by the stimulation frequency: a higher stimulation frequency elicited a larger-magnitude displacement. We used the stimulation frequency as the manipulated variable in our closed-loop control system, and the controlled leg was successfully set at predetermined angular positions. In conclusion, coupled with the low power consumption compared with that of entirely man-made legged robots, the ability to regulate a beetle's leg motion under a closed-loop control system should contribute significantly to the future design of biological actuators and hence biological legged machines (i.e., insect–machine hybrid legged robots). Note that the leg-muscle configurations are common or similar (i.e., a pair of antagonistic muscle groups dominating the leg displacement in opposite directions) among many insect orders. In addition, various stimulation protocols to elicit leg displacement in the desired direction have been proposed and demonstrated for various insect orders, including moth, stick insect, and locust [22]-[24]. Therefore, the methodology and experimental design demonstrated in this paper may be applicable to the development of a closed-loop control of the leg motion of other insect orders.

## Supporting Information

Table S1 **Mean and standard deviation of overshoot angle (Degree) with respect to different Kp and update time interval values.**
(DOCX)

Table S2 **Mean and standard deviation of reaching time (s) with respect to different Kp and update time interval values.**
(DOCX)

Table S3 **Mean and standard deviation of power consumption rate (µW) for all the six muscle groups of the beetle's front leg across five beetles.**
(DOCX)

Video S1 **Demonstration of leg motion control, as shown in** **Figure 9** **.** The four LEDs indicate the electrical stimulation times and periods. Each motion could either be individually elicited or simultaneously coupled with other motion using isolated stimulation channels.
(MOV)
