# Supplementary material for: A Biological Micro Actuator: Graded and Closed-Loop Control of Insect Leg Motion by Electrical Stimulation of Muscles
Source: PLoS One. 2014 Aug 20;9(8):e105389. doi: 10.1371/journal.pone.0105389 (PMC4139336; doi:10.1371/journal.pone.0105389)
Supplement: Table S1 — Mean and standard deviation of overshoot angle (Degree) with respect to different Kp and update time interval values. (DOCX) [file pone.0105389.s001.docx]

|  |  | **Update time interval (ms)** | | |
| --- | --- | --- | --- | --- |
|  | **Kp** | **100** | **200** | **300** |
| **Protraction** | 0.5 | 10.47 ± 3.66 | 10.82 ± 11.02 | 12.09 ± 12.34 |
|  | 1.0 | 17.51 ± 5.79 | 16.10 ± 7.99 | 16.01 ± 8.39 |
|  | 1.5 | 22.12 ± 7.75 | 19.91 ± 4.99 | 18.74 ± 9.98 |
| **Retraction** | 0.5 | 11.03 ± 5.06 | 6.13 ± 4.29 | 5.05 ± 3.60 |
|  | 1.0 | 15.97 ± 5.38 | 10.82 ± 4.44 | 6.52 ± 4.34 |
|  | 1.5 | 17.48 ± 4.94 | 13.47 ± 4.76 | 10.77 ± 4.31 |
