# Supplementary material for: A Biological Micro Actuator: Graded and Closed-Loop Control of Insect Leg Motion by Electrical Stimulation of Muscles
Source: PLoS One. 2014 Aug 20;9(8):e105389. doi: 10.1371/journal.pone.0105389 (PMC4139336; doi:10.1371/journal.pone.0105389)
Supplement: Table S2 — Mean and standard deviation of reaching time (s) with respect to different Kp and update time interval values. (DOCX) [file pone.0105389.s002.docx]

|  |  | **Update time interval (ms)** | | |
| --- | --- | --- | --- | --- |
|  | **Kp** | **100** | **200** | **300** |
| **Protraction** | 0.5 | 0.518 ± 0.133 | 0.776 ± 0.228 | 0.949 ± 0.328 |
|  | 1.0 | 0.350 ± 0.184 | 0.436 ± 0.086 | 0.564 ± 0.150 |
|  | 1.5 | 0.299 ± 0.188 | 0.368 ± 0.174 | 0.403 ± 0.161 |
| **Retraction** | 0.5 | 1.249 ± 0.917 | 1.479 ± 0.829 | 2.225 ± 1.514 |
|  | 1.0 | 0.492 ± 0.217 | 0.771 ± 0.479 | 0.875 ± 0.308 |
|  | 1.5 | 0.488 ± 1.111 | 0.510 ± 0.194 | 0.692 ± 0.342 |
