# Supplementary material for: A Biological Micro Actuator: Graded and Closed-Loop Control of Insect Leg Motion by Electrical Stimulation of Muscles
Source: PLoS One. 2014 Aug 20;9(8):e105389. doi: 10.1371/journal.pone.0105389 (PMC4139336; doi:10.1371/journal.pone.0105389)
Supplement: Table S3 — Mean and standard deviation of power consumption rate (µW) for all the six muscle groups of the beetle's front leg across five beetles. (DOCX) [file pone.0105389.s003.docx]

|  | **Five beetles with weight (g)/ length (cm) shown** | | | | |
| --- | --- | --- | --- | --- | --- |
| **Muscle group** | 8.47 g/ 6.5 cm | 6.06 g/ 5.3 cm | 7.76 g/ 5.5 cm | 6.57 g/ 5.9 cm | 10.35 g/ 6.7 cm |
| Protraction | 182.34 ± 2.25 | 123.16 ± 10.15 | 126.54 ± 4.44 | 133.68 ± 0.72 | 135.22 ± 19.15 |
| Retraction | 136.70 ± 6.56 | 101.55 ± 3.89 | 197.48 ± 16.15 | 128.70 ± 0.72 | 94.62 ± 1.59 |
| Depression | 137.67 ± 1.05 | 72.42 ± 1.92 | 202.81 ± 9.56 | 173.96 ± 3.78 | 119.88 ± 0.42 |
| Levation | 151.85 ± 1.61 | 176.00 ± 3.20 | 200.34 ± 1.92 | 136.05 ± 2.87 | 139.62 ± 4.36 |
| Extension | 125.75 ± 1.40 | 114.84 ± 3.73 | 164.40 ± 1.71 | 227.05 ± 9.55 | 144.18 ± 3.33 |
| Flexion | 163.29 ± 4.72 | 120.07 ± 1.50 | 149.77 ± 6.73 | 210.96 ± 3.38 | 154.18 ± 6.37 |
